# Pathway networks generated from human disease phenome

**DOI:** 10.1186/s12920-018-0386-2

**Published:** 2018-09-14

**Authors:** Ann G. Cirincione, Kaylyn L. Clark, Maricel G. Kann

**Affiliations:** Department of Biological Sciences, University of Maryland, Baltimore County (UMBC), Baltimore, MD 21250 USA

**Keywords:** Networks, Phenome, Disease mutations

## Abstract

**Background:**

Understanding the effect of human genetic variations on disease can provide insight into phenotype-genotype relationships, and has great potential for improving the effectiveness of personalized medicine. While some genetic markers linked to disease susceptibility have been identified, a large number are still unknown. In this paper, we propose a pathway-based approach to extend disease-variant associations and find new molecular connections between genetic mutations and diseases.

**Methods:**

We used a compilation of over 80,000 human genetic variants with known disease associations from databases including the Online Mendelian Inheritance in Man (OMIM), Clinical Variance database (ClinVar), Universal Protein Resource (UniProt), and Human Gene Mutation Database (HGMD). Furthermore, we used the Unified Medical Language System (UMLS) to normalize variant phenotype terminologies, mapping 87% of unique genetic variants to phenotypic disorder concepts. Lastly, variants were grouped by UMLS Medical Subject Heading (MeSH) identifiers to determine pathway enrichment in Kyoto Encyclopedia of Genes and Genomes (KEGG) pathways.

**Results:**

By linking KEGG pathways through underlying variant associations, we elucidated connections between the human genetic variant-based disease phenome and metabolic pathways, finding novel disease connections not otherwise detected through gene-level analysis. When looking at broader disease categories, our network analysis showed that large complex diseases, such as cancers, are highly linked by their common pathways. In addition, we found Cardiovascular Diseases and Skin and Connective Tissue Diseases to have the highest number of common pathways, among 35 significant main disease category (MeSH) pairings.

**Conclusions:**

This study constitutes an important contribution to extending disease-variant connections and new molecular links between diseases. Novel disease connections were made by disease-pathway associations not otherwise detected through single-gene analysis. For instance, we found that mutations in different genes associated to Noonan Syndrome and Essential Hypertension share a common pathway. This analysis also provides the foundation to build novel disease-drug networks through their underlying common metabolic pathways, thus enabling new diagnostic and therapeutic interventions.

**Electronic supplementary material:**

The online version of this article (10.1186/s12920-018-0386-2) contains supplementary material, which is available to authorized users.

## Background

Current repositories of disease-associated human genetic variants encompass over 4000 genes and 17,000 disease phenotypes, derived mostly from manual extraction of literature [1]. Our team has compiled these variants from sources including the Online Mendelian Inheritance in Man (OMIM) [2], Clinical Variance database (ClinVar) [3], Universal Protein Resource (UniProt) [4], and Human Gene Mutation Database (HGMD) [1, 5]. Our compilation contains over 80,000 human genetic variants and is the largest collection known to date [1]. Yet despite the large number of genetic variants known to influence disease, only a small percentage of an individual genome is expected to map to *known* variants. To identify novel disease-variant associations, the functional context of known variants must be expanded.

In prior studies, human disease networks (e.g., the human diseasome) have been generated to link genetic disorders with disease genes, supporting disease-specific functional modules [6]. Genes rarely work alone, often participating in complex pathways and synergic interactions with other genes, proteins, and environmental factors that collectively influence the clinical manifestations of disease [7–9]. Analyzing known disease-associated variants through a network biology approach can provide insight into the relationship between diseases and underlying metabolic pathways, and can expand the functional context of our variants [10]. This may result in novel disease associations not otherwise discovered through single-gene analysis. Existing research has shown that exploring genetic risk through a modular approach allows for more stable and robust results and improves the interpretation of molecular mechanisms underlying disease [8].

The first challenge in identifying disease-pathway associations from known variants is to correctly assign each variant phenotype to an ontology. A standardized terminology provides an accurate mapping of diseases to disease-specific categories and facilitates the identification of enriched pathway associations. Thus, the first step in this work was to map variant phenotypes to the Unified Medical Language System (UMLS) [11] to build a disease phenome. The UMLS [11] leverages the UMLS Metathesaurus to integrate a wide variety of phenotype synonyms from terminologies like OMIM [2] and the Human Phenotype Ontology (HPO) [12]. Once variants were mapped, they were clustered into higher-level categories via Medical Subject Heading (MeSH) identifiers, providing a broader view of the variant relationships.

To identify disease-pathway associations, mapped variants were linked at both the disease and pathway level. Our work builds on the approach taken in Goh et al. [6], expanding gene-based connections to their corresponding networks of human variant-derived Kyoto Encyclopedia of Genes and Genomes (KEGG) [13] pathways. Network representations were used, illustrating the power of our approach by providing a more complete view of human disease-variant associations. Our networks produced over 1300 novel disease associations at the pathway level, not otherwise connected at the gene level. This provides potential new insight into the relationship between metabolic pathways and interacting drugs, and may lead to improved drug efficacy and new potential drug targets for repurposing. [14] These associations will constitute an important resource in the future development of computational tools to optimize patient diagnosis and disease treatment.

## Methods

### Mapping human variants to UMLS

We mapped the Peterson et al. [1] compilation of over 80,000 human disease variants, which unifies specific genetic data from databases including OMIM [2], ClinVar [3], UniProt [4], and HGMD [5], to disorder concepts in the UMLS Metathesaurus. This mapping was performed using exact and normalized string matching functions from the UMLS Terminology Server (UTS) Application Program Interface (API). Variants were categorized by two different classifications: primarily by Concept Unique Identifier (CUI) and secondarily by MeSH. Each specific CUI categorizes all disease phenotypes equivalent to the same concept, linking those that may use a different phenotype terminology but ultimately define the same entity [11]. MeSH provides a hierarchical set of descriptors, allowing for a controlled vocabulary and a broader level of specificity when searching for disease terms [11]. Both CUI and MeSH classifications will allow human variants to be clustered based on their disease descriptions, providing an important link between genotype and phenotype.

Human variants unable to be mapped automatically to UMLS [11] were manually curated. Curators used the UMLS Metathesaurus browser to search unique phenotypes and variations of their terminology after removing modifiers and other fragments deemed unessential to the concept. Phenotypes were then classified into three types, per the amount of manual manipulation necessary to find a matching CUI (Additional file 1: Figure S1). Variants categorized as Type I were matched with little to no modification; Type II categorization required a moderate amount of manipulation; and Type III represents variant phenotypes unable to be mapped. Once completed, this manual mapping will allow for Type I and Type II variants to be mapped to a higher-order MeSH classification, increasing the number of variants in our further analysis at the pathway level.

### Functional enrichment of variants

Mapped human variants were clustered into higher-level disease categories based on MeSH terms. For each grouping, genes mutated in the variants mapped to that MeSH were statistically enriched in KEGG pathways (one-tailed Fisher exact test, *p* < = 0.0001) for groups associated with at least three genes. MeSH categories enriched in at least three pathways were selected for network construction.

### Network construction

**Fig. 1:**
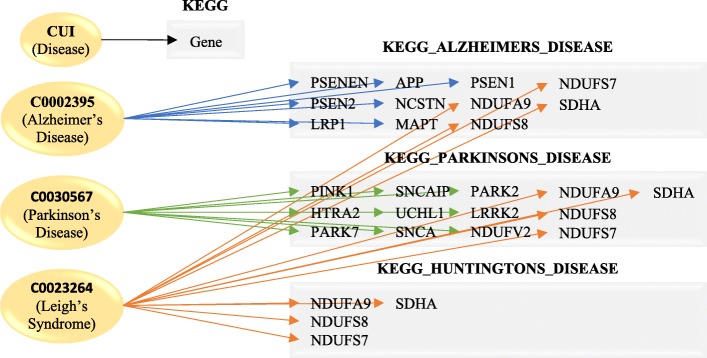
Human Disease Phenome construction. A bipartite graph was constructed, linking diseases to pathways. Each CUI consists of multiple genes, which are enriched in KEGG pathways (*p* < = 0.0001). One CUI can link to multiple pathways, and one pathway can be linked to multiple CUIs

A bipartite graph was constructed using two disjoint sets of nodes: CUIs and KEGG Pathways (Fig. 1). Each distinct disease, identified by its UMLS CUI, is linked to a KEGG pathway if it included genes statistically enriched in that pathway. Networks linking CUIs were generated using Cytoscape [15]. In these networks, each node represented a CUI, and two CUIs were connected if they shared at least one common KEGG pathway. Additionally, networks linking variants on a pathway level were generated. For these networks, each node represented a KEGG pathway, and two pathways were connected if they shared at least one common CUI enriched in those pathways. Topical analysis was performed using the Network Analyzer Cytoscape plugin [15]. Separate networks for each MeSH category were generated, with each node shaded a various color based on both its mapping to MeSH and how exclusive the mapping was. Lastly, all networks of unique MeSH term pairings were compared for statistically significant overlap of enriched KEGG pathways, with selected pairs chosen for comparison network construction.

## Results

### Mapping of variants to a phenotype ontology

**Fig. 2:**
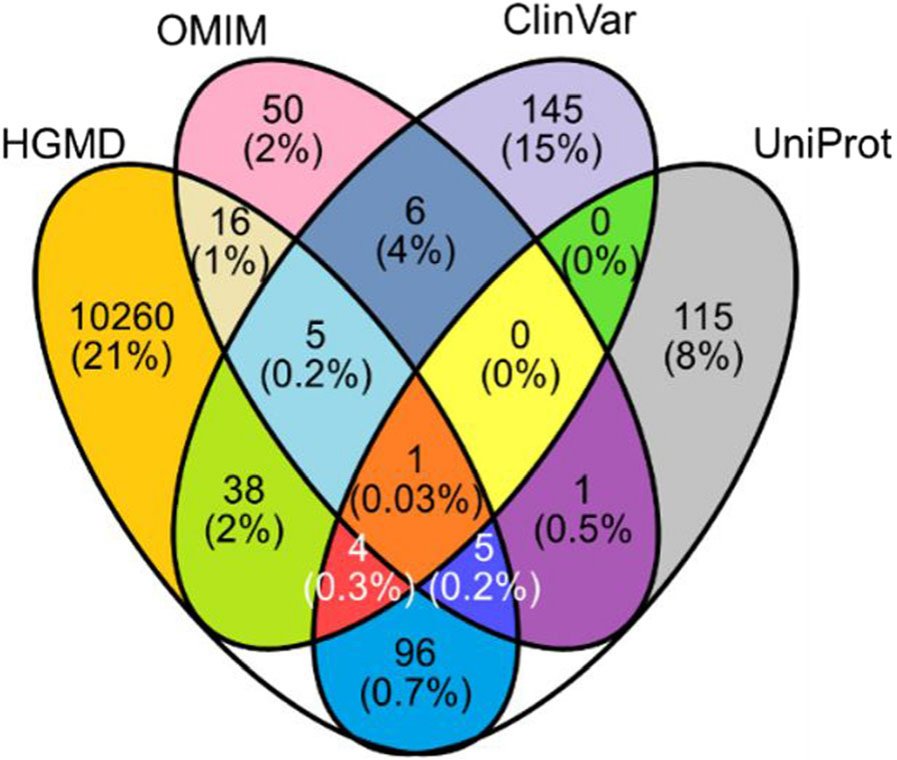
Fraction of human variants [1] not mapped to UMLS [11], separated by source database. Number of variants unable to be mapped, as a percent of total variants curated in each specific source database combination

Through UTS API functions, 87% of variants were computationally mapped. Out of 80,686 variants in our database, those unable to be mapped originated from different source databases (Fig. 2). A manual curation protocol was developed to rescue unique phenotypes otherwise unable to be mapped to UMLS [11]. Currently, 56% of variants unable to be computationally mapped have been manually curated, with 16% classified as Type I, 23% as Type II, and 61% as Type III.

### Pathway enrichment of variants and construction of networks by MeSH term

**Fig. 3:**
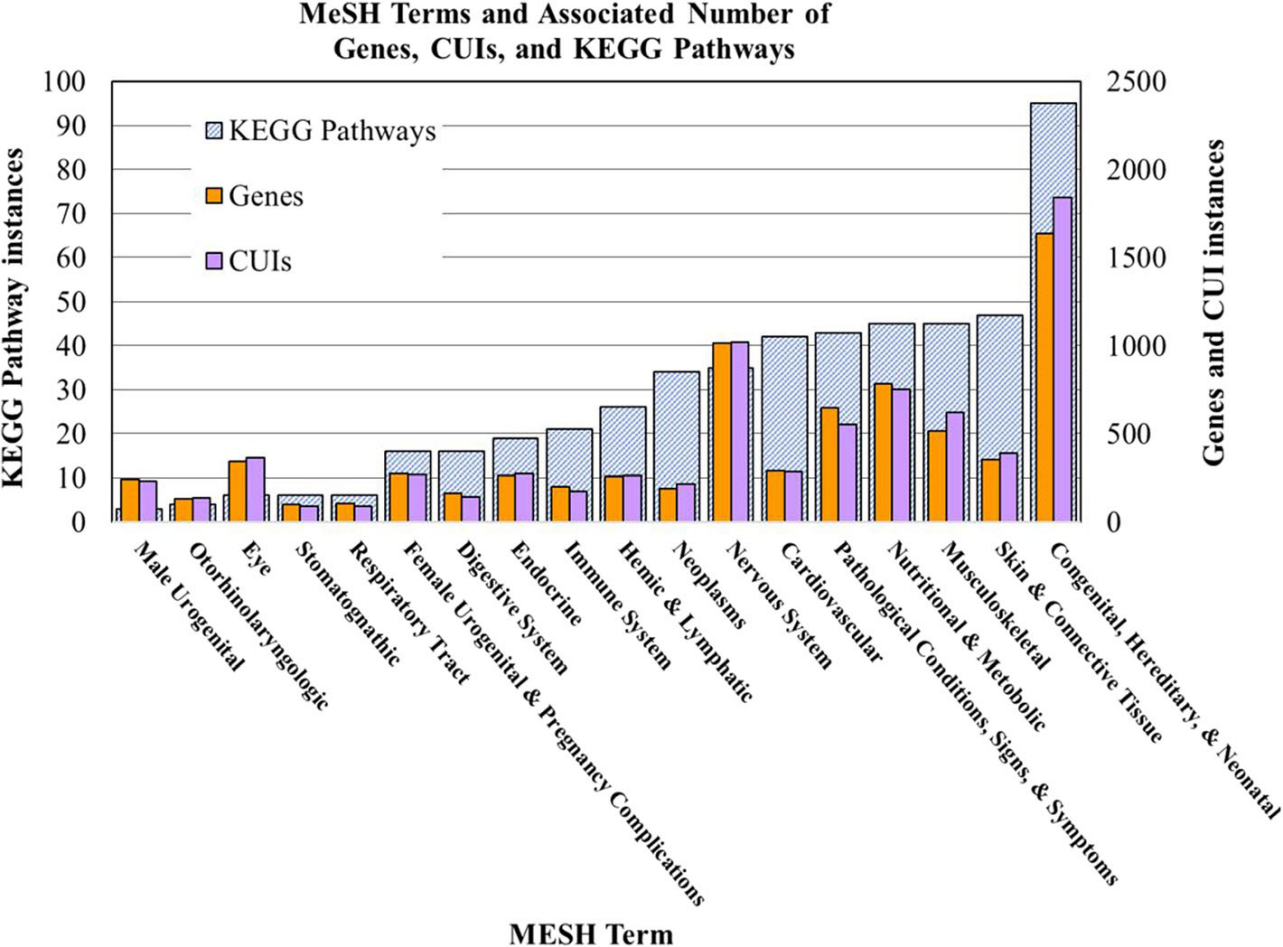
Relative number of genes, CUIs, and KEGG pathways associated with each MeSH term. Categorizations of human variants based on MeSH classifications were enriched in KEGG pathways (*p* < = 0.0001). Groups with at least three enriched pathways were analyzed

Human variants were grouped based on MeSH to provide a higher-level phenotypic categorization. MeSH term groupings varied in number of associated genes, CUIs, and KEGG pathways (Fig. 3). Groups were associated with between 98 and 1636 genes, 88–1839 CUIs, and 3–95 KEGG pathways (Fig. 3). The MeSH category with the largest number of enriched KEGG pathways was Congenital, Hereditary, and Neonatal Diseases and Abnormalities (C16), with 95 pathways enriched (79% of all pathways enriched in MeSH categories combined); (Fig. 3). Additionally, 69% of human variants were mapped to this MeSH category. This is likely because most known, disease-associated human genetic variants were compiled from databases that encompass inherited diseases. For more specific MeSH terms, such as Stomatognathic Diseases (C07), fewer KEGG pathways were enriched. This is likely because only a small number of genes have a known association to this disease in our repository.

**Fig. 4:**
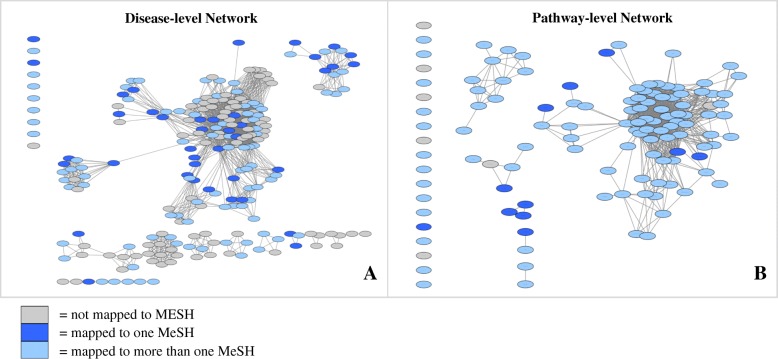
Network graphs showing connections between genetic disorders at the disease (CUI) and pathway (KEGG) levels. Out of 223 CUIs statistically enriched in at least one KEGG pathway (*p* < = 0.0001), 213 (96%) can be connected to other CUIs through a common pathway (**a**). Out of 121 KEGG pathways, 109 (90%) can be connected to other pathways through a common CUI (**b**). Both graphs are shaded according to the number of MeSH categories to which each node maps

**Fig. 5:**
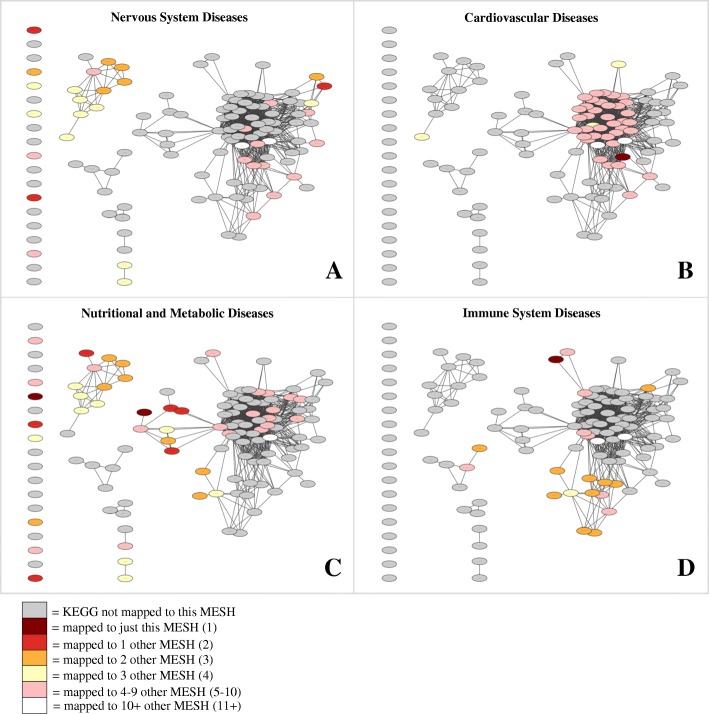
Networks linking genetic disorders to KEGG pathways colored by four separate MeSH terms. Nodes represent KEGG pathways, connected through underlying links between common CUIs. Networks are colored by MeSH term, specifically Nervous System Diseases (**a**), Cardiovascular Diseases (**b**), Nutritional and Metabolic Diseases (**c**), and Immune System Diseases (**d**)

Separate networks were generated to illustrate the link between human genetic diseases: one at the disease level (Fig. 4a), and one at the pathway level (Fig. 4b). Out of 223 CUIs in the disease-level network, 90 (40%) were not mapped to MeSH, 40 (18%) were mapped to a single MeSH, and 93 (42%) were mapped to more than one MeSH term. Out of 121 KEGG pathways in the pathway-level network, 7 (6%) were not mapped to MeSH, 11 (9%) were mapped to a single MeSH, and 103 (85%) were mapped to more than one MeSH term. Simple parameters of both disease and pathway level networks were determined using the Network Analyzer Cytoscape plugin [15] (Table 1). The pathway-based network was also illustrated using separate networks for each individual MeSH term. Four of these networks are illustrated in Fig. 5, shaded based on how exclusive each mapping is. For example, dark red shading distinguishes the most exclusive pathways, to which only a single MeSH is mapped. In contrast, white shading distinguishes the least exclusive pathways, to which between 11 and 18 MeSH terms are mapped.

**Table 1:** Simple parameter network analysis [15] of disease-level and pathway-level networks

	Disease-level Network	Pathway-level Network
Number of nodes	213	102
Number of edges	2079	857
Clustering coefficient	0.835	0.722
Connected components	15	6
Network diameter	4	5
Shortest paths	19,514 (43%)	6302 (61%)
Characteristic path length	2.322	2.029
Avg. number of neighbors	19.521	16.804
Network density	0.092	0.166
Network heterogeneity	1.042	0.961

The network parameters were calculated from connected nodes only (with at least one edge)

**Fig. 6:**
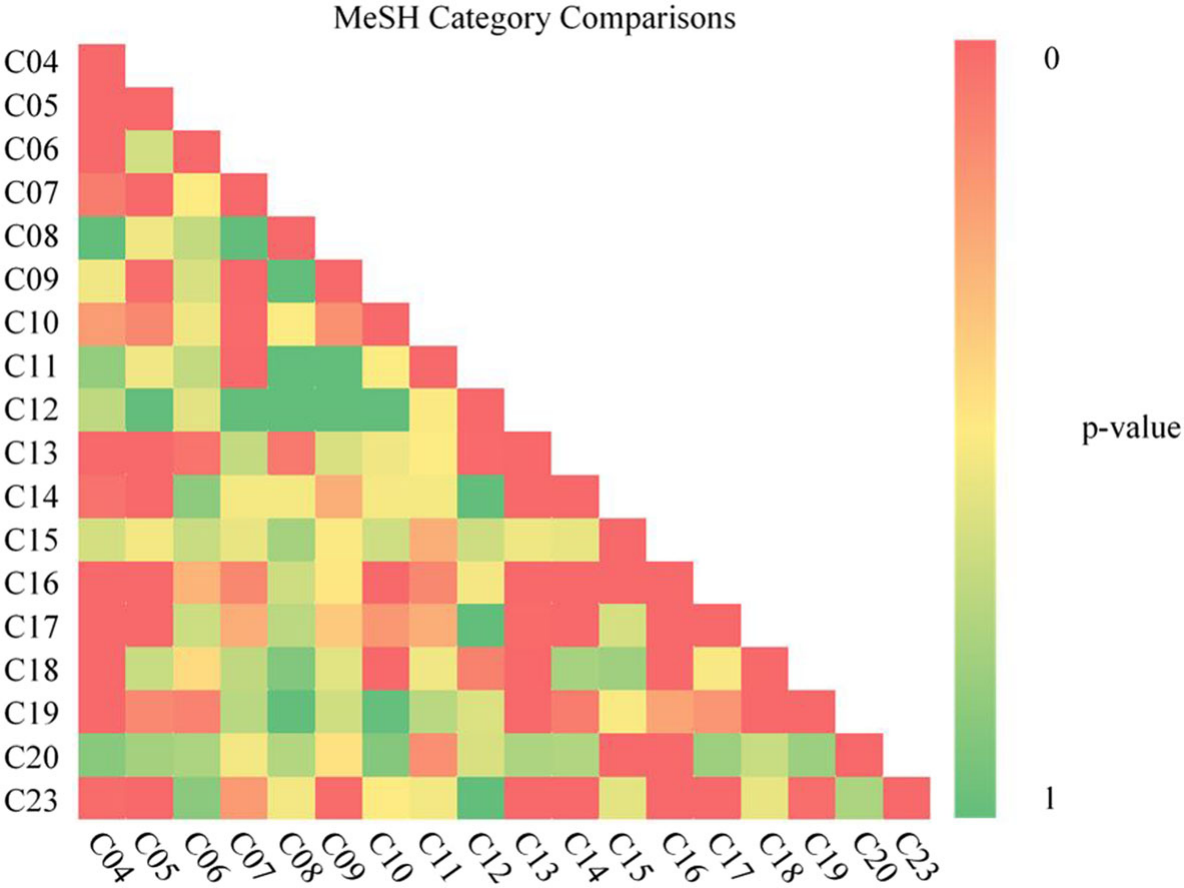
Pairwise comparison of MeSH categories. For each of the 135 MeSH pairs, *p*-values denote the significance of the number of common pathways. C04 = Neoplasms (D009369); C05 = Musculoskeletal Diseases (D009140); C06 = Digestive System Diseases (D004066); C07 = Stomatognathic Diseases (D009057); C08 = Respiratory Tract Diseases (D012140); C09 = Otorhinolaryngologic Diseases (D010038); C10 = Nervous System Diseases (D009422); C11 = Eye Diseases (D005128); C12 = Male Urogenital Diseases (D052801); C13 = Female Urogenital Diseases and Pregnancy Complications (D005261); C14 = Cardiovascular Diseases (D002318); C15 = Hemic and Lymphatic Diseases (D006425); C16 = Congenital, Hereditary, and Neonatal Diseases and Abnormalities (D009358); C17 = Skin and Connective Tissue Diseases (D017437); C18 = Nutritional and Metabolic Diseases (D009750); C19 = Endocrine System Diseases (D004700); C20 = Immune System Diseases (D007154) and C23 = Pathological Conditions, Signs and Symptoms (D013568)

A total of 153 combinations of unique MeSH term pairings were compared, with 35 (23%) exhibiting a significant overlap (*p* < = 0.001) of enriched KEGG pathways (Fig. 6, Additional file 1: Table S1). The pairing between Cardiovascular Diseases and Skin and Connective Tissue Diseases had the highest overlap of 39 common pathways (*p* = 6E-32).

**Fig. 7:**
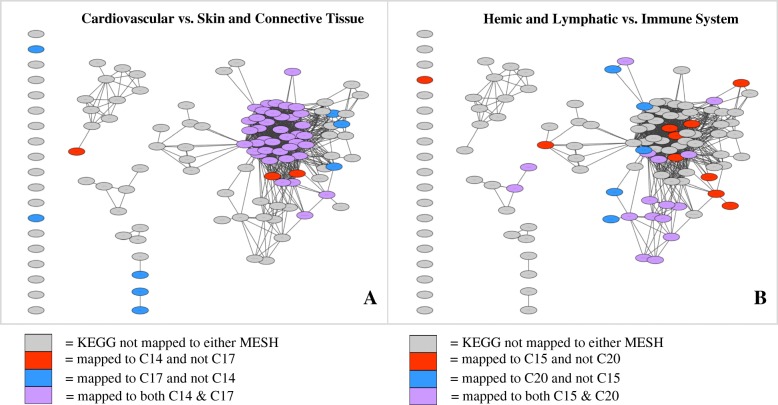
Comparison networks between two separate MeSH term categories. Intersection between C14 and C17 networks (**a**) as well C15 and C20 networks (**b**) yield statistically significant overlap (*p* = 1E-28, *p* = 1E-12, respectively). KEGG pathways colored according to MeSH categorization. (see abbreviations in Fig. 5)

When comparing unique MeSH term combinations, two significant pairings included Cardiovascular Diseases (C14) versus Skin and Connective Tissue Diseases (C17), and Hemic and Lymphatic Diseases (C15) versus Immune System Diseases (C20); (Fig. 7). For each pairing, KEGG pathways were colored with respect to which MeSH categories they were enriched in. For example, in the first pairing of C14 vs. C17 in Fig. 7a, nodes enriched in only C14 are colored red, those enriched in only C17 are colored blue, and those enriched in both C14 and C17 are colored purple. Out of a total of 51 pathways enriched in either C14 or C17, 39 (76%) are enriched in both. Additionally, in Fig. 7b, out of a total of 32 pathways enriched in either C15 and C20, 16 (50%) are enriched in both.

## Discussion

Using UMLS string matching algorithms, we mapped the Peterson et al. [1] collection of human variants to the UMLS [11] phenotype ontology and built a collection of almost 70,000 (87%) unique human genetic variants to disorder concepts. UMLS [11] offers wide coverage of variants and higher-level disease categorization through MeSH. This allowed us to cluster phenotypes into broader categories and make inferences about each subgroup. Unique genetic variants associated with phenotypes otherwise unable to be mapped to UMLS [11] were run through a manual curation protocol, through which 56% of variants have currently been manually curated. Mapping to UMLS [11] allowed human variants to be grouped together based on same and/or similar phenotype, alleviating many of the difficulties faced due to the lack of standardization of vocabulary.

Disease-level and pathway-level bipartite networks were constructed using KEGG pathway enrichment, linking human genetic variants by common pathways or CUIs. In both networks, one central cluster of nodes was the most highly connected. The nodes in this cluster generally encompassed pathways involved in essential processes (i.e., reproduction, survival), many of which may be altered in and/or directly linked to cancer. [10] Through network analyzation [15] of the pathway-level network, KEGG *Pathways in Cancer* was found to have a high connectivity in the central cluster, with the highest node degree of 52 (Table 1).

The disease-level network contained 223 CUIs connected by 2548 unique disease associations through gene- or pathway-level analysis. Of these, 1338 (53%) connections are only observed through disease-pathway associations and not otherwise connected at the gene-level. Additionally, 461 (18%) connections are observed through disease-gene associations only, and 741 (29%) connections are observed through both gene- and pathway-level associations. When CUIs are connected through both common genes and common pathways, the resulting disease associations function as confidence builders with a higher level of evidence to support the connection. Hypoglycaemia, hyperinsulinaemic (C1864903) and Diabetes, type 2 (C0011860) were connected through the genes HNF1A, ABCC8, HNF4A, and GCK, as well as the KEGG pathway *Type II Diabetes Mellitus*. This association is expected, as hypoglycemia is known to affect type II diabetes patients near insulin-deficiency [16]. CUI connections made through pathways but not through genes extend the functional context of variants and provide new potential disease associations. Noonan Syndrome (C0028326) and Essential Hypertension (C0085580) were connected through the KEGG pathway *Vascular Smooth Muscle Contraction*, despite associated variants not having any common genes in our repository. A common symptom of Noonan syndrome is hypertrophic cardiomyopathy [17], which in turn is highly related to hypertension and often occurs in conjunction in elderly patients [18], suggesting a logical connection between Noonan Syndrome and Essential Hypertension concepts in our network.

As shown in Fig. 7, comparison of Cardiovascular (C14) and Skin and Connective Tissue (C17) networks shows high overlap in the largest cluster of KEGG pathways, which includes basic cellular functions such as cell signaling, growth, and maintenance. Many of these kinds of pathways are also altered in different types of cancer, as seen by the connections and enrichment of cancerous pathways in the main network cluster. A few examples include *Melanoma, MAPK Signaling Pathway,* and *Pathways in Cancer.* The high similarity between C14 and C17 is to be expected, as many cardiac disorders involve the connective tissue within/surrounding the heart, and relationships have been observed between normal development of connective tissue and the cardiovascular system [19]. Comparison of Hemic and Lymphatic (C15) and Immune System (C20) networks shows high overlap in a cluster of immunological KEGG pathways, including *Primary Immunodeficiency*, *Type I Diabetes Mellitus*, and *Autoimmune Thyroid Disease*. This intersection is also expected to be significant, as lymphatic diseases are highly linked to the immune surveillance and adaption [20].

Our next step is to continue analyzing human genetic variants at different levels of clustering, expanding our classifications and extending functional context to find new disease connections. If a pathway is found to link to multiple diseases, a drug being used to treat one disease could potentially be repurposed to treat another disease connected at the same pathway level [7]. In addition, if a disease is found to link to multiple pathways, a patient with this disease may benefit from a pathway-guided combination therapy [7]. With the addition of patient data, variant-based disease-pathway associations can be compared across individuals and provide a platform for incorporating new variant data into our database. In the future, this will allow us to develop computational tools that facilitate the optimization of personalized diagnosis, prognosis, and disease treatment.

## Conclusions

Expanding our view of the human diseasome to include human variant-derived KEGG pathway associations allowed for an extended functional view of disease-variant associations. Novel disease connections were made by disease-pathway associations not otherwise detected through single-gene analysis. This shows that seemingly unrelated disease variants can be associated at the pathway level. Additionally, this type of analysis provides new relationships between metabolic pathways and disease-drug networks, potentially enabling novel diagnostic and therapeutic interventions.

## Additional file

Additional file 1: **Figure S1.** detailing manual curation protocol and **Table S1.** providing MeSH term pairings. (DOCX 27 kb)

## Abbreviations

API: Application Program Interface
ClinVar: Clinical variance database
CUI: Concept unique identifier
HGMD: Human gene mutation database
HPO: Human phenotype ontology
KEGG: Kyoto encyclopedia of genes and genomes
MeSH: Medical Subject Heading
OMIM: Online mendelian inheritance in man
UMLS: Unified medical language system
UniProt: Universal protein resource
UTS: UMLS terminology server
