# Supplementary material for: Pathway networks generated from human disease phenome
Source: BMC Med Genomics. 2018 Sep 14;11(Suppl 3):75. doi: 10.1186/s12920-018-0386-2 (PMC6156841; doi:10.1186/s12920-018-0386-2)
Supplement: Supplementary file 1 — Figure S1. detailing manual curation protocol and Table S1. providing MeSH term pairings. (DOCX 27 kb) [file 12920_2018_386_MOESM1_ESM.docx]

**Pathway Networks Generated From Human Disease Phenome**

**Supplemental Material**

**Type I**

**Type II**

**Type III**

- CUI found from exact phenotype
- CUI found for slightly more specific phenotype

Example
Original: *Cone-rod synaptic disorder*
Found: *Cone-rod synaptic disorder, congenital nonprogressive*

- One CUI found, may or may not be the same disease
- Multiple CUIs found, fairly certain which one is true
- CUI found for more generalized phenotype

Example
Original: *Calcified aortic valve with ascending aortic aneurysm*
Found: *Calcified aortic valve*

- No CUIs found at all
- Multiple CUIs found, not sure which (if any) is correct
- No hits from full phenotype, multiple hits when searching fragments
- CUI found, but most likely not the same disease

Example
Original: *Congenital myasthenic syndrome, limb-girdle*

**Figure S1.** **Manual curation protocol categorization of unique phenotypes otherwise unable to be mapped to UMLS.** Variants were categorized as type I, II, or III, increasing with the amount manipulation needed to match to a CUI. Examples of variants from our database are included for reference.

| **MeSH Categories** | **Common Pathways (p-value)** |
| --- | --- |
| C14, C23 | 39 (6E-32) |
| C14, C17 | 39 (1E-28) |
| C05, C14 | 38 (7E-28) |
| C05, C17 | 39 (8E-26) |
| C17, C23 | 38 (1E-25) |
| C05, C23 | 37 (6E-25) |
| C16, C17 | 47 (1E-17) |
| C05, C16 | 45 (8E-17) |
| C16, C23 | 42 (4E-14) |
| C14, C16 | 41 (1E-13) |
| C20, C15 | 16 (1E-12) |
| C16, C10 | 34 (7E-11) |
| C04, C16 | 32 (4E-09) |
| C18, C16 | 39 (1E-08) |
| C19, C13 | 10 (5E-08) |
| C07, C09 | 4 (3E-07) |
| C19, C18 | 14 (1E-06) |
| C16, C15 | 24 (2E-06) |
| C04, C05 | 19 (8E-06) |
| C04, C13 | 10 (4E-05) |
| C04, C19 | 11 (4E-05) |
| C14, C13 | 11 (4E-05) |
| C13, C23 | 11 (5E-05) |
| C16, C20 | 19 (7E-05) |
| C05, C13 | 11 (9E-05) |
| C18, C13 | 11 (9E-05) |
| C05, C07 | 6 (0.00015) |
| C16, C13 | 15 (0.00021) |
| C04, C18 | 17 (0.00025) |
| C11, C07 | 3 (0.00037) |
| C04, C06 | 9 (0.00038) |
| C18, C10 | 17 (0.00040) |
| C04, C17 | 17 (0.00049) |
| C12, C13 | 3 (0.00053) |
| C07, C10 | 5 (0.00096) |

**Table S1. Significant comparisons of unique MeSH term pairings.**

MeSH categories exhibiting a significant overlap (p <= 0.001) of enriched KEGG pathways with respective number of pathways in common.
